# An intact complement system dampens cornea inflammation during acute primary HSV-1 infection

**DOI:** 10.1038/s41598-021-89818-9

**Published:** 2021-05-13

**Authors:** Adrian Filiberti, Grzegorz B. Gmyrek, Amanda N. Berube, Derek J. Royer, Daniel J. J. Carr

**Affiliations:** 1 Departments of Ophthalmology, The University of Oklahoma Health Sciences Center (OUHSC), 608 Stanton L. Young Blvd., DMEI PA415, Oklahoma City, OK. 73104 USA; 2 Microbiology and Immunology, The University of Oklahoma Health Sciences Center, Oklahoma City, OK 73104 USA

**Keywords:** Antimicrobial responses, Chemokines, Complement cascade, Cytokines, Infection, Inflammation, Innate immune cells, Innate immunity, Lymphatic system, Mucosal immunology, Cell biology, Immunology, Diseases, Pathogenesis

## Abstract

Corneal transparency is an essential characteristic necessary for normal vision. In response to microbial infection, the integrity of the cornea can become compromised as a result of the inflammatory response and the ensuing tissue pathology including neovascularization (NV) and collagen lamellae destruction. We have previously found complement activation contributes to cornea pathology-specifically, denervation in response to HSV-1 infection. Therefore, we investigated whether the complement system also played a role in HSV-1-mediated neovascularization. Using wild type (WT) and complement component 3 deficient (C3 KO) mice infected with HSV-1, we found corneal NV was accelerated associated with an increase in inflammatory monocytes (CD11b^+^CCR2^+^CD115^+/−^Ly6G^−^Ly6C^high^), macrophages (CD11b^+^CCR2^+^CD115^+^Ly6G^−^Ly6C^high^) and a subpopulation of granulocytes/neutrophils (CD11b^+^CCR2^−^CD115^+^Ly6G^+^Ly6C^low^). There were also increases in select pro-inflammatory and pro-angiogenic factors including IL-1α, matrix metalloproteinases (MMP)-2, MMP-3, MMP-8, CXCL1, CCL2, and VEGF-A that coincided with increased inflammation, neovascularization, and corneal opacity in the C3 KO mice. The difference in inflammation between WT and C3 KO mice was not driven by changes in virus titer. However, viral antigen clearance was hindered in C3 KO mouse corneas suggesting the complement system has a dynamic regulatory role within the cornea once an inflammatory cascade is initiated by HSV-1.

## Introduction

HSV-1 is a significant human pathogen that can result in progressive corneal damage leading to visual impairment^1^. In experimental models of HSV-1 keratitis, the host inflammatory response to the pathogen is the principal driver of tissue pathology which includes neovascularization (NV)^2^, lymphangiogenesis^3^, collagen fiber disorientation^4^, infiltration of leukocytes^1^, neurotrophic keratitis^5,6^, and edema^7^ that can result in scarring and opacity of the cornea^8^. Whereas this process typically occurs following a single infectious episode under experimental conditions in mice, it is often experienced in the human host after multiple bouts of virus reactivation from latency^9^. Steroids applied in a time-dependent fashion have been found to diminish some ocular manifestations of experimental HSV-1 keratitis^10,11^ and reduce the persistence or progression of stromal inflammation in human patients^12^.

The innate immune system is rapidly activated following HSV-1 corneal infection consisting of resident hematopoietic-derived and non-hematopoietic-derived cells that express sensors to detect HSV-1 nucleic acid and proteins. Toll-like receptors (TLR) including TLR-2, TLR-4, and TLR-9, upon activation, are reported to elicit corneal inflammation and lesion development but do not directly contribute to NV^13–16^. Another innate sensor, interferon inducible protein 16 (IFI-16), through the activation of the stimulator of interferon (IFN) genes (STING) drives epithelial cell expression of type I IFN and tetherin that control HSV-1 replication and dissemination^17,18^. Resident plasmacytoid dendritic cells and macrophages within the cornea are also involved in the early innate response to control virus replication and inflammation through the production of soluble factors and antigen clearance^19,20^. Likewise, mast cells that reside adjacent to the vasculature in the limbus respond rapidly to HSV-1 infection of the cornea and contribute to the early control of virus replication^21^. Another component of the innate immune system that serves as a sentinel surveillance pathway and rapidly responds to microbial insult and assists the innate and adaptive immune systems in the clearance of pathogens is complement. The activation of the complement system (CS) occurs within seconds of a pathogen insult enhancing phagocytosis of complement-laden pathogen-associated antigens or pathogens (e.g., virus), contributing to antibody neutralization, facilitating disruption of pathogen integrity through the development of the membrane attack complex, and assisting in the attraction of macrophages and neutrophils^22^. As a result of the potent inflammatory potential of the CS^23^, spontaneous activation of the CS in the normal cornea is inhibited by several complement regulatory proteins including, membrane cofactor protein (CD46), decay-acceleration factor (CD55), surface regulator of complement, and membrane inhibitor of reactive lysis (CD59)^24^.

HSV-1 encodes a number of immune evasion molecules that disrupt innate and adaptive immune system processes^25,26^. In terms of complement, HSV-1 glycoprotein C binds to human complement component 3 (C3) and prevents complement-mediated neutralization of virus and lysis of infected cells^27,28^. Within the cornea, C3 has been found to contribute to HSV-1-mediated denervation^29^. As C3 is associated with the recruitment and activation of innate immune cells^30^ of which a subpopulation of CD115^+^ myeloid-derived cells have been found to be instrumental in HSV-1-elicited NV and lymphangiogenesis^31^, we reasoned the absence of a functional CS would diminish the severity of virus-mediated NV and lymphangiogenesis. In contrast with this hypothesis, we found corneal NV and opacity to be accelerated in C3 deficient (C3 KO) mice that correlated with an elevated inflammatory response including an increase in select myeloid cell subpopulations and inflammatory/pro-angiogenic factor expression in a time-dependent fashion. Thus, the results suggest a protective role of C3 delaying corneal inflammation and NV following HSV-1 infection.

## Results

### C3 KO mice succumb to ocular HSV-1 infection associated with an increase in virus loads in the nervous system compared to wild type mice

**Figure 1:**
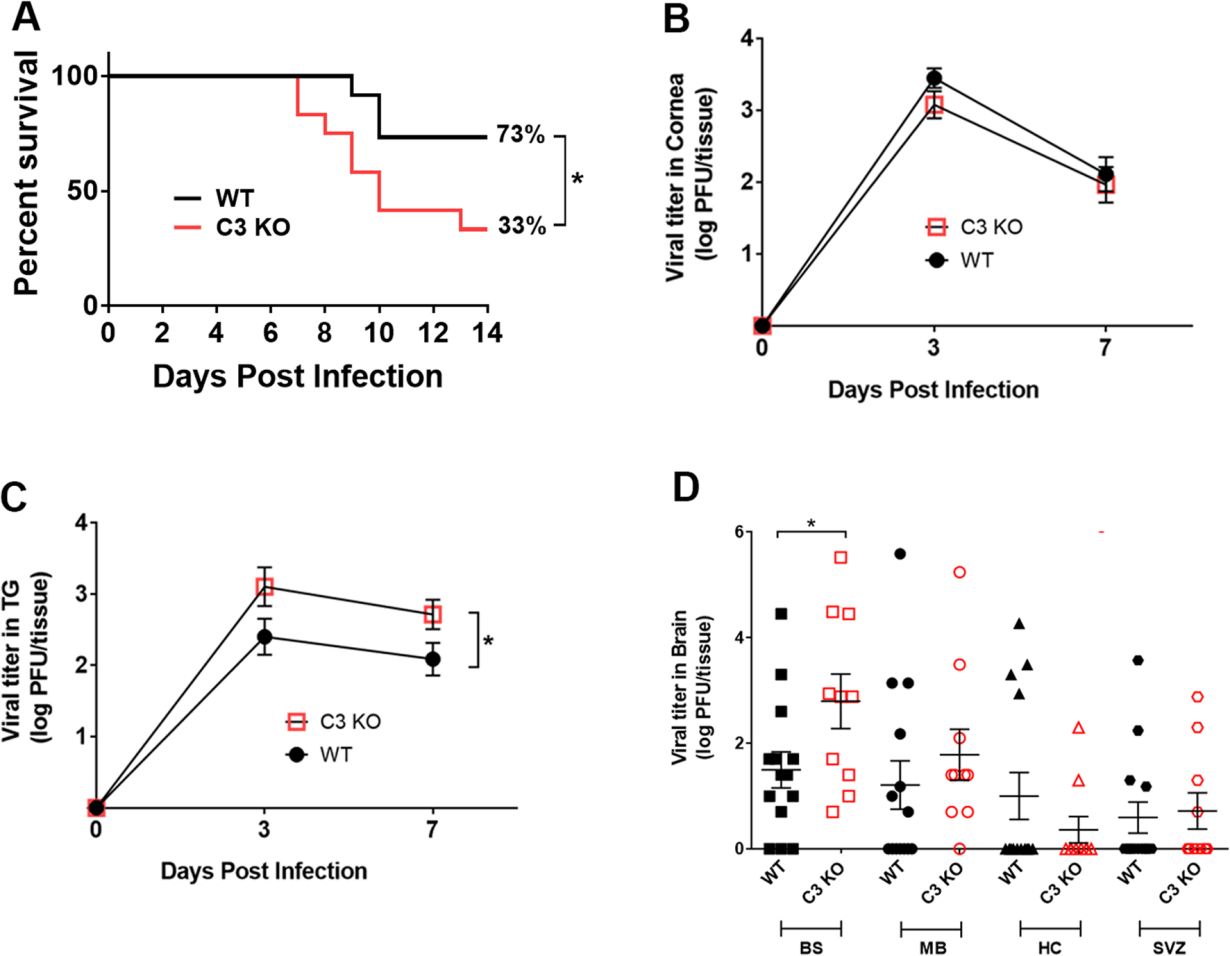
C3 KO mice mortality is elevated following ocular HSV-1 infection. WT and C3 KO male and female mice were infected with 500 PFU (HSV-1)/cornea. (**A**) WT and C3 KO mice (n = 30/group) were monitored for survival over 14 days pi. *p < 0.05 as determined by Longrank and Gehan–Breslow–Wilcoxon test, Chi-square distribution. Viral titer quantification in cornea (**B**) and trigeminal ganglia (**C**) of WT and C3 KO mice (n = 6/group/time point) were determined by plaque assay at 0, 3, and 7 days pi. Horizontal bars depict mean of log PFU/tissue ± SEM. *p < 0.05 as determined by unpaired multiple t-test with Holm–Sidak correction. (**D**) Viral titers were determined by plaque assay in brain regions including brain stem (BS), midbrain (MB), hippocampus (HC), and subventricular zone (SVZ) at day 7 pi. Horizontal bars depict mean of log PFU/tissue ± SEM. *p < 0.05 as determined by unpaired multiple t-test with Holm-Sidak correction, n = 10–14 samples/group/tissue.

The CS is critical in the maintenance and balance of immune homeostasis through a strong association with innate defense against microbial pathogen infection^32^. Consistent with this idea, we found ocular challenge with HSV-1 results in a significant decrease in survival in C3 KO mice compared to wild type (WT) control animals (Fig. 1A). Although there was no difference in viral titers recovered from the cornea of WT and C3 KO mice at days 3 or 7 post infection (pi) (Fig. 1B), there was a significant increase in the amount of infectious virus recovered from the trigeminal ganglia (TG) of C3 KO mice compared to that recovered from WT TG at day 7 but not day 3 pi (Fig. 1C). HSV-1 is known to traffic to the central nervous system during acute infection and establish latency^33,34^. With this in mind, we assessed the spread of HSV-1 throughout the brain and found C3 KO mice were found to possess significantly more infectious virus in the brain stem compared to WT mice at day 7 pi following ocular administration (Fig. 1D). However, there was no difference in viral titer from other regions of the brain including the midbrain, hippocampus, or subventricular zone (Fig. 1D). Taken together, the results suggest the CS contributes directly to viral surveillance in the peripheral and central nervous system during acute primary infection that leads to the demise of mice following ocular virus infection.

### The incidence of pathology is accelerated in the cornea of C3 KO mice following HSV-1 infection

**Figure 2:**
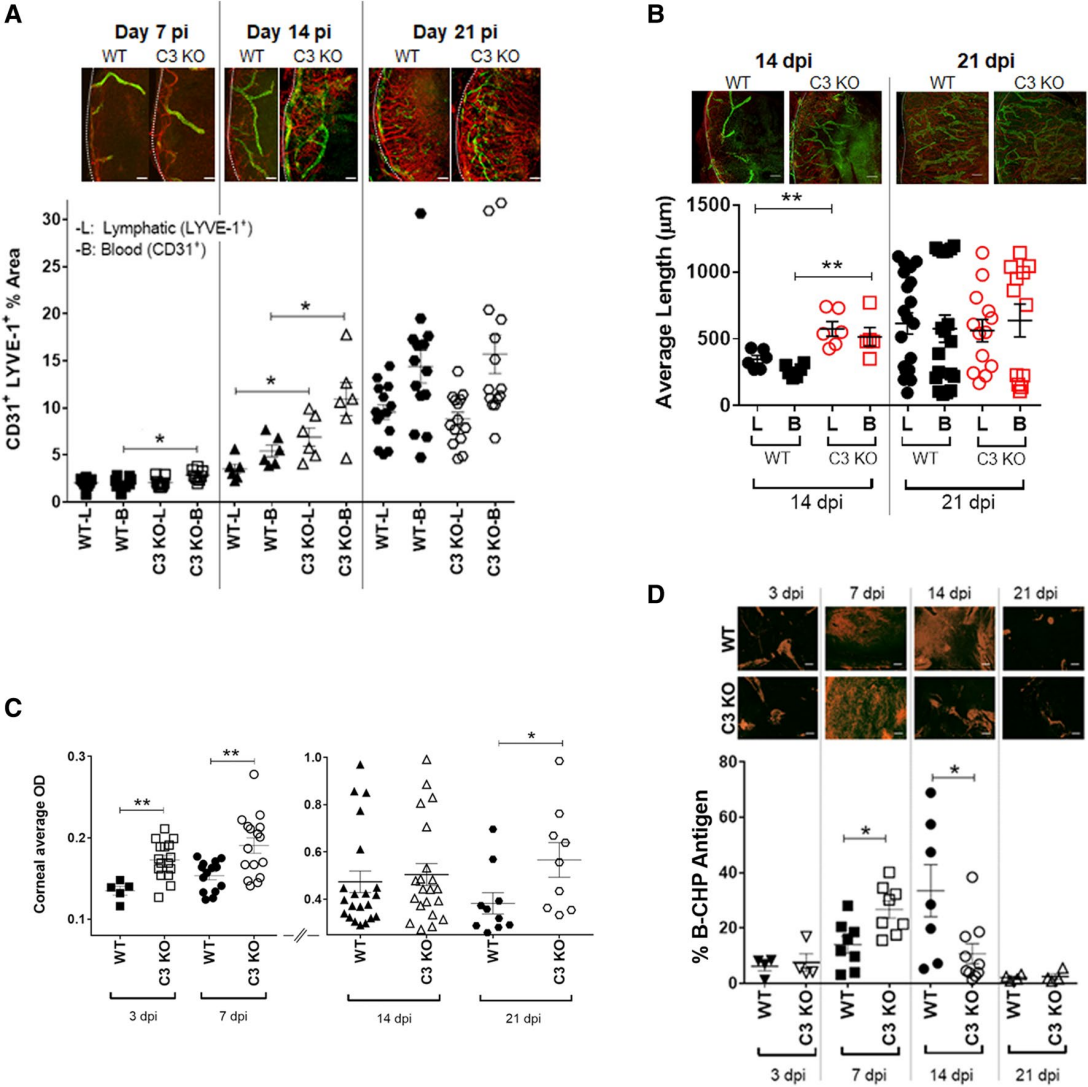
Acceleration in ocular pathology is observed in C3 KO mice following ocular HSV-1 infection. WT and C3 KO male and female mice were infected with 500 PFU (HSV-1)/cornea. (**A**) Corneal neovascularization quantification of WT and C3 KO mouse corneas at 7, 14 and 21 days pi. Top panel, representative confocal microscopy images of flat whole mount cornea preparations from 7 to 21 days pi. Red: blood vessels (CD31^**+**^), green: lymphatic vessels (LYVE-1^**+**^). Horizontal bars depict mean percentage threshold of the *z*-stacked area positive for blood and lymphatic vessels ± SEM. *p < 0.05 comparing the indicated groups as determined by unpaired, two-tailed t-test comparison, n = 5 per treatment, two repetitions. Discontinuous white lines represent the corneoscleral limbus margin. (**B**) Length measurement of blood and lymphatic vessels at 14 and 21 days pi. Top panel, representative confocal microscopy images of flat whole mount cornea preparations at 14 and 21 dpi. Red: blood vessels (CD31^**+**^), green: lymphatic vessels (LYVE-1^**+**^). Horizontal bar depict mean of average length measured from the corneoscleral limbus to the center of the cornea (centripetal development) ± SEM. **p < 0.01 comparing the indicated groups as determined by unpaired, two-tailed *t*-test, n = 5 per treatment, two repetitions. (**C**) Opacity of WT and C3 KO mouse corneas was measured at the indicated time point (3, 7, 14, or 21 days pi) by optical density (OD) using a plate reader at 500-nm wavelength in 30 × 30 matrix distributed over the cornea surface. Horizontal bars depict mean of OD average ± SEM. **p < 0.001 comparing the indicated groups as determined by unpaired, two-tailed t-test comparison, n = 5 per mouse genotype, two repetitions. (**D**) Collagen disruption of WT and C3 KO mouse corneas measured at the indicated time point (3, 7, 14 or 21 days pi). Top panel, representative confocal microscopy images of flat whole mount cornea samples showing denatured collagen stained with collagen hybridizing peptide biotin conjugated (B-CHP, orange). Horizontal bars depict the mean of percent *z*-stacked area positive for B-CHP ± SEM. *p < 0.05 comparing the indicated groups as determined by unpaired, two-tailed t-test comparison, n = 4–8/treatment, two repetitions. Scale bar: 100 μm.

HSV-1 elicits a robust innate immune response in the mouse cornea following infection including neovascularization and lymphangiogenesis^35,36^. Laser-induced choroidal neovascularization has been tied to C3, as C3 KO mice do not develop choroidal neovascularization in response to laser injury^37^. Consequently, we assessed the presentation of WT and C3 KO mice following HSV-1 infection using spectral domain-optical coherence tomography (SP-OCT) and found the corneas of the C3 KO mice to show a trend for greater edema than their WT counterparts at day 5 and 7 pi (Supplementary Fig. 1S). However, consistent with previous results^29^ the C3 KO mice maintained mechanosensory function which was lost by WT mice measured using a Cochet Bonnet esthesiometer (Supplementary Fig. 1S). Accordingly, increased myeloid inflammation in the corneas of C3 KO mice corresponds with exacerbated inflammation of the ocular adnexa during HSV-1 infection. To further compare cornea pathology between WT and C3 KO mice, we investigated the genesis of blood and lymphatic vessels in the cornea over time in response to HSV-1 infection. As early as day 7 pi, there was a modest but significant difference in the blood vessel genesis in the cornea with more ingrowth of CD31^+^ vessels observed in the HSV-1 C3 KO mouse cornea (Fig. 2A). This difference became more pronounced by day 14 pi and now included lymphatic vessels (Fig. 2A). Furthermore, the lymph and blood vessels extended further into the cornea of C3 KO mice compared to the WT animals at this time point (Fig. 2B). By day 21 pi, the blood and lymphatic vessels occupied equivalent overall area of the cornea comparing WT and C3 KO mice, and the length of the WT vessels had caught up to that found in the cornea of C3 KO mice (Fig. 2B).

Similar to neovascularization/lymphangiogenesis, there was an acceleration in opacity development in the C3 KO mice relative to WT animals (Fig. 2C). Specifically, whereas there was no difference in cornea opacity at day 1 pi (data not shown); however, by day 3 pi there was a significant increase in opacity in the corneas of C3 KO mice relative to WT animals that remained elevated through day 7 pi (Fig. 2C). By day 14 pi, the opacity of the HSV-1-infected WT and C3 KO mice were similar (Fig. 2C). However, by day 21 pi, the opacity in the cornea of WT mice began to resolve whereas that found in the C3 KO animals remained (Fig. 2C).

A number of factors contribute to corneal opacity and maintenance of transparency including keratocyte phenotype and corneal stroma collagen fiber diameter and spacing^38,39^. To determine whether collagen remodeling/architecture may be a contributing factor in the opacity observed in these corneas, we employed a biotinylated-collagen hybridizing peptide that selectively binds to denatured collagen strands^40^. In HSV-1-infected WT mice, collagen fiber denaturation was first detected at day 3 pi, peaked at day 14 pi, and resolved by day 21 pi (Fig. 2D). By comparison, in C3 KO mice collagen remodeling was found to occur at a similar level to WT mice at day 3 pi, peak at day 7 pi, and begin to resolve at day 14 pi (Fig. 2D). Thus, corneal opacity in C3 KO mice peaked significantly sooner than that found in WT mice with similar times of resolution at day 21 pi. However, the observation that collagen fiber denaturation was no longer evident by day 21 pi suggests the opacity observed in the C3 KO mice is not due to denatured collagen lamellae at the latter time point but rather, likely due to additional soluble factors and/or cells as well as neovascularization.

### Pro-inflammatory and pro-angiogenic factors are elevated in the cornea of C3 KO mice in a time-dependent fashion

**Figure 3:**
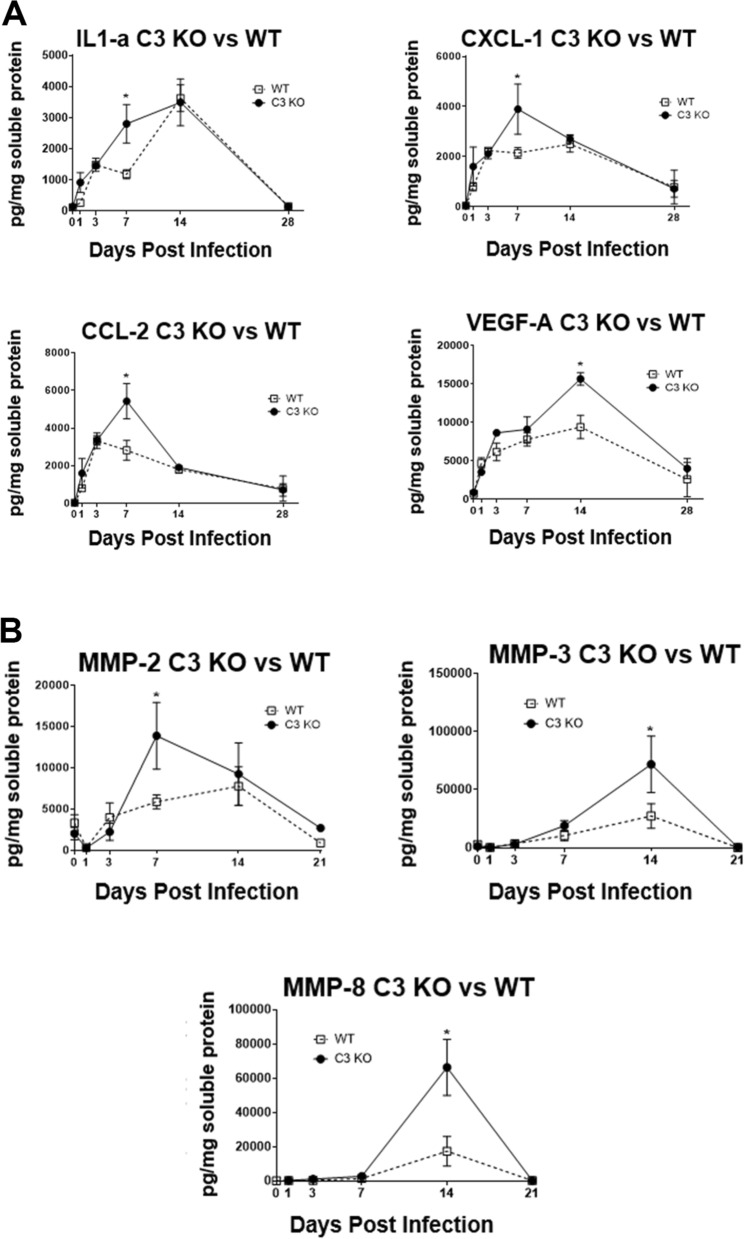
The absence of C3 temporally enhances MMP and cytokine/chemokine expression in the cornea. HSV-1 infected WT and C3 KO mice (n = 6/group/time point) were euthanized by exsanguination at the indicated time pi. The corneas were removed and processed for analyte assessment by multiplex suspension array. (**A**) Levels of IL-1α, CXCL1, CCL2, and VEGF-A and (**B**) MMP-2, MMP-3, and MMP-8 are shown for the indicated time points. Each time point is displayed as the mean pg/mg protein ± SEM representing the summary of two experiments. *p < 0.05 comparing the two groups for the indicated time point as determined by unpaired *t*-test with Holm–Sidak correction.

Soluble factors known to be expressed during ocular HSV-1 infection that contribute directly or indirectly to neovascularization or reduced transparency include but are not limited to VEGF-A, FGF-2, IL-1α, IL-6, CXCL1, and CCL2 as well as matrix metalloproteinases (MMP)^41–44^. In response to HSV-1 infection, the pro-angiogenic factors including IL-1α, CXCL1, CCL2, and VEGF-A were found to be elevated in the cornea of C3 KO mice in a time-dependent fashion with IL-1α, CXCL1, and CCL2 levels significantly increased in the cornea of C3 KO mice at day 7 pi whereas VEGF-A levels were significantly higher at day 14 pi compared to WT animals (Fig. 3A). IL-6 and FGF-2 levels were elevated but were not significantly different in the C3 KO mice compared to the WT animals (data not shown). Likewise, MMP2 levels were significantly elevated in the cornea of HSV-1-infected C3 KO mice at day 7 pi whereas MMP3 and MMP8 concentrations were significantly increased at day 14 pi (Fig. 3B). Taken together, the rise in select pro-inflammatory and pro-angiogenic factors in the cornea of C3 KO mice is consistent with the increase in tissue pathology (i.e., neovascularization and opacity) observed and likely contributes to these events.

### Myeloid cell infiltration in C3 KO mice increases above WT mice in response to HSV-1 infection

**Figure 4:**
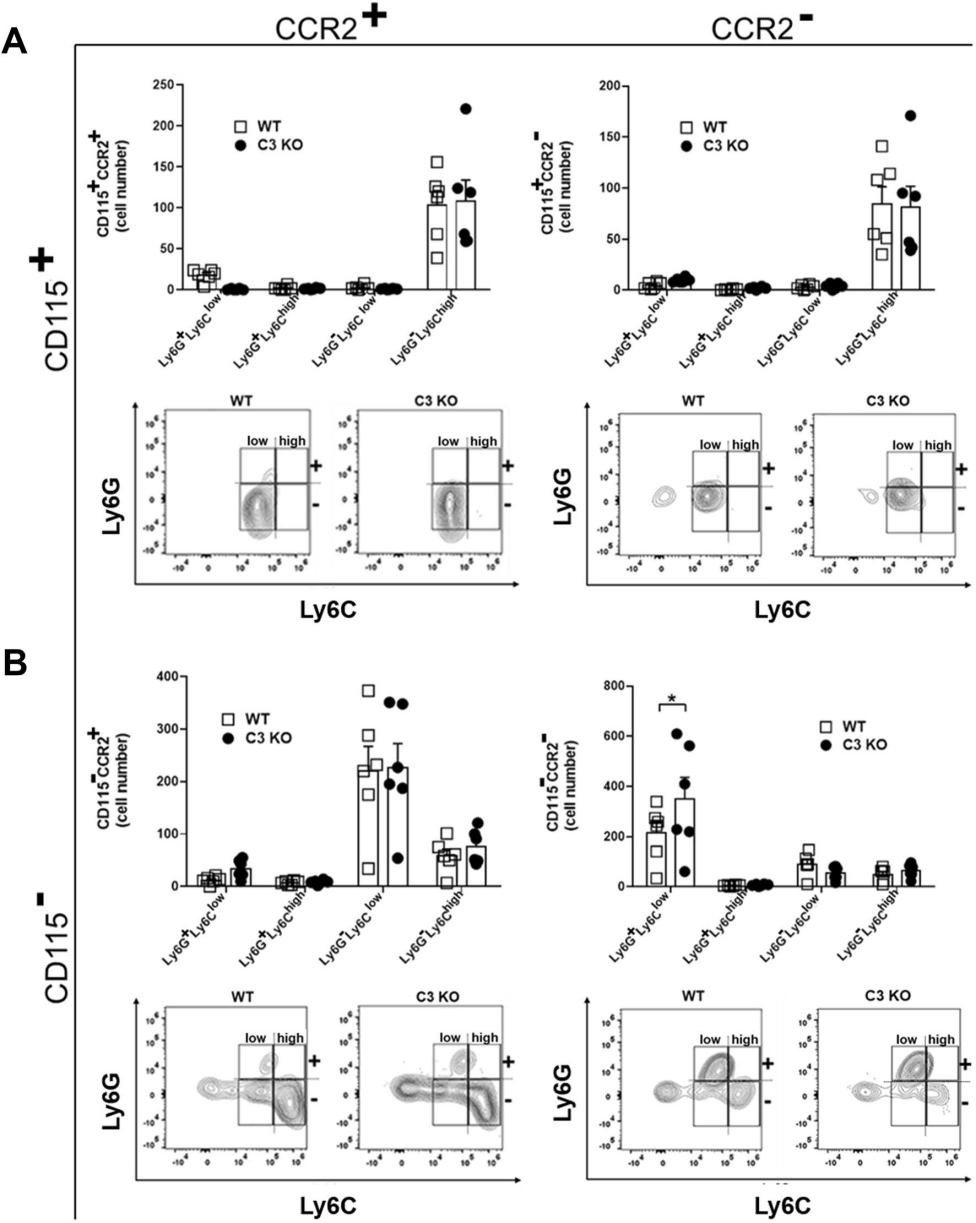
A subpopulation of granulocytes/neutrophils are elevated in the cornea of C3 KO mice at day 3 post infection. HSV-1 infected C3 KO and WT mice were euthanized by exsanguination at 3 days pi, and the corneas were removed and enzymatically processed to single cell suspensions. (**A**) Absolute number of CD45^+^CD11b^+^CD115^+^ Ly6G and Ly6C myeloid cell subpopulations that do or do not express CCR2. Panels below are representative flow plots of cell distribution in terms of high- and low-expressing Ly6G and Ly6C phenotypes. (**B**) Absolute number of CD45^+^CD11b^+^CD115^−^ Ly6G and Ly6C myeloid cell subpopulations that do or do not express CCR2. Panels below are representative flow plots of cell distribution in terms of high- and low-expressing Ly6G and Ly6C phenotypes. *p < 0.05 comparing the C3 KO to WT mouse CD45^+^CD11b^+^Ly6G^+^Ly6C^low^CCR2^−^CD115^−^ granulocyte/neutrophil phenotype as determined by unpaired, two-tail *t*-test. The results displayed are from two independent experiments.

**Figure 5:**
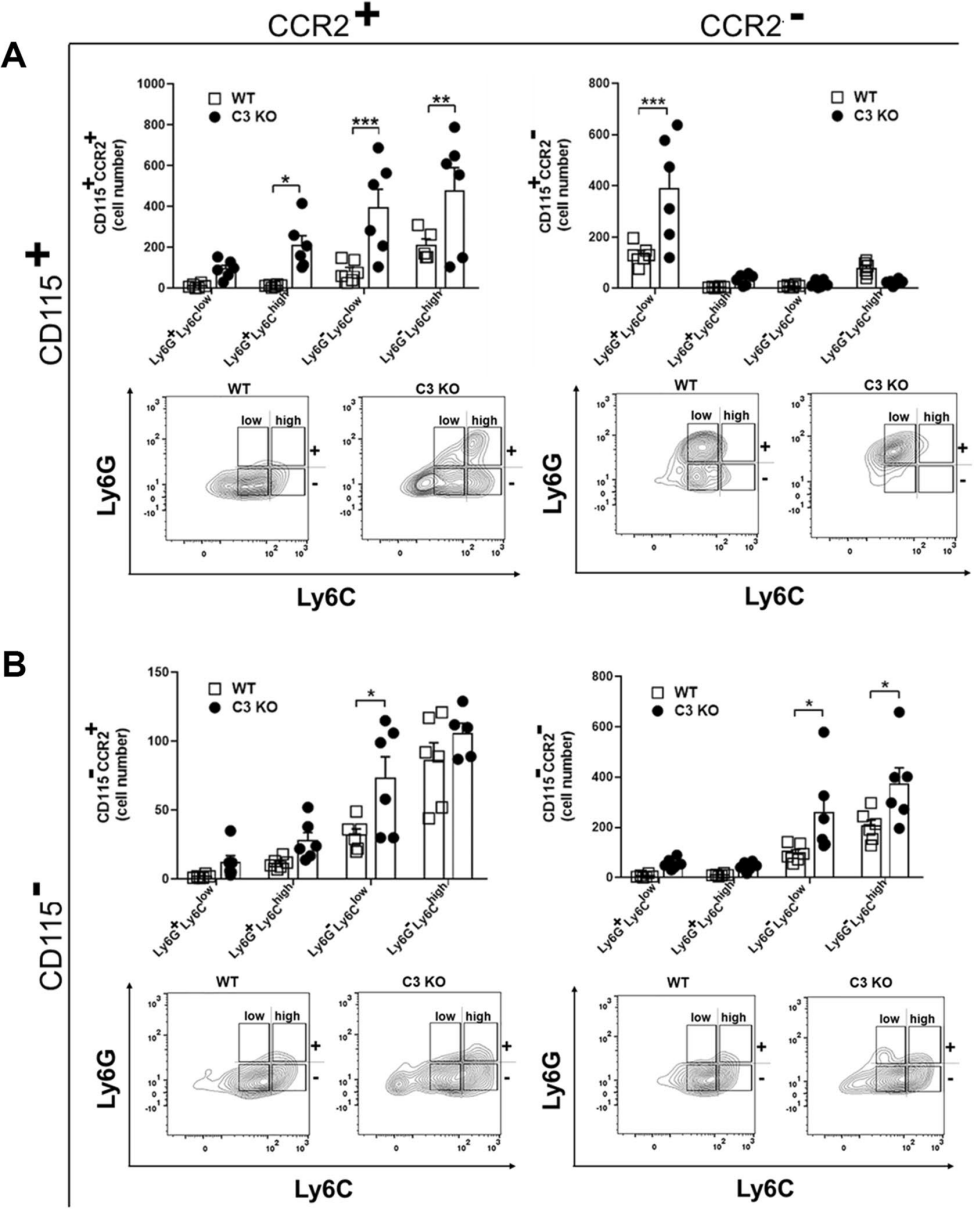
Multiple myeloid-derived cell populations are elevated in the cornea of C3 KO mice at day 7 post infection. HSV-1 infected C3 KO and WT mice were euthanized by exsanguination at 7 days pi, and the corneas were removed and enzymatically processed to single cell suspensions. (**A**) Absolute number of CD45^+^CD11b^+^CD115^+^ Ly6G and Ly6C myeloid cell subpopulations that do or do not express CCR2. Panels below are representative flow plots of cell distribution in terms of high- and low-expressing Ly6G and Ly6C phenotypes. (**B**) Absolute number of CD45^+^CD11b^+^CD115^−^ Ly6G and Ly6C myeloid cell subpopulations that do or do not express CCR2. Panels below are representative flow plots of cell distribution in terms of high- and low-expressing Ly6G and Ly6C phenotypes. ***p < 0.001, **p < 0.01, and *p < 0.05 comparing the C3 KO to WT mouse indicated phenotypes of granulocytes/neutrophils, inflammatory monocytes, and macrophages as determined by unpaired, two-tail *t*-test. The results displayed are from two independent experiments.

**Figure 6:**
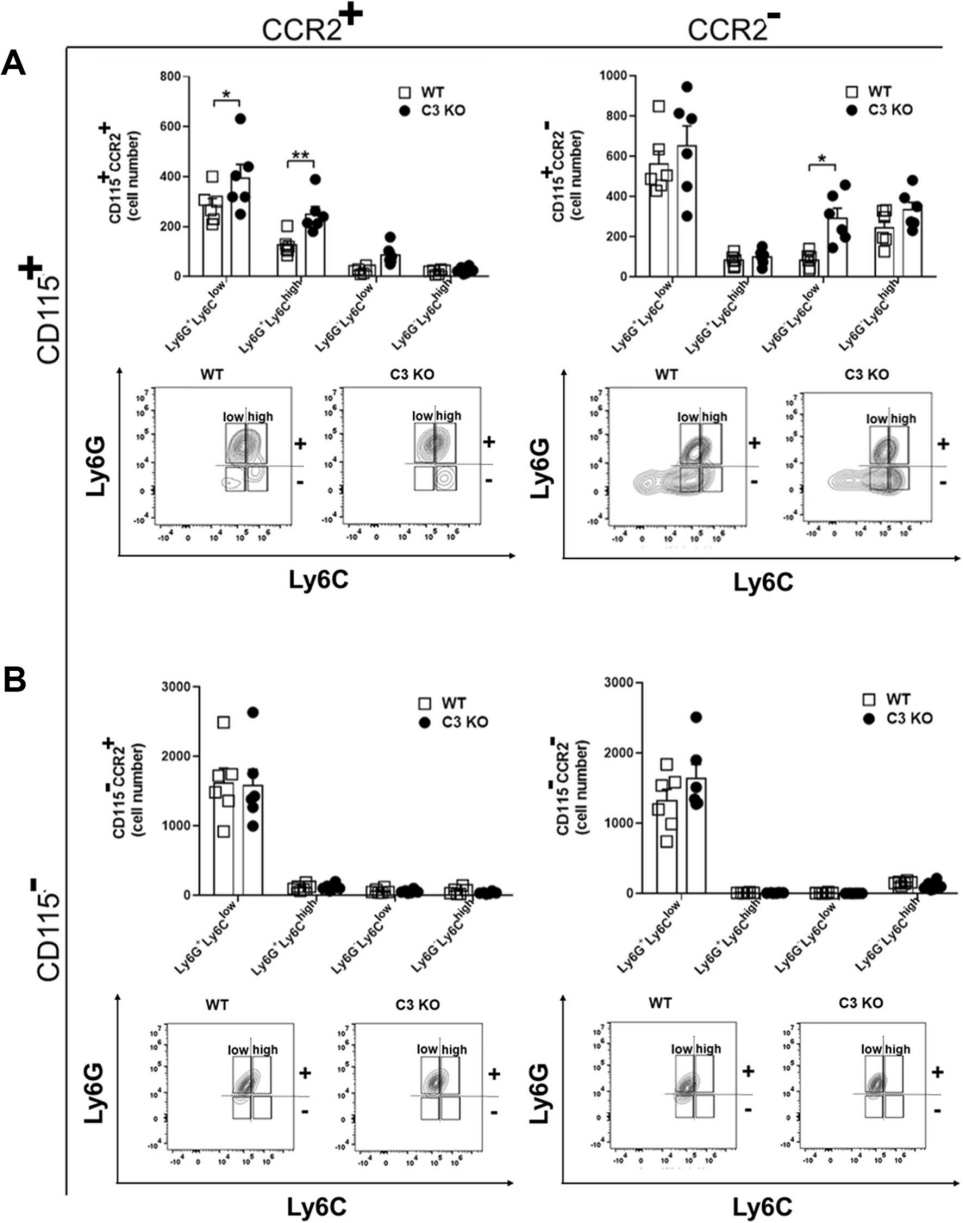
A subpopulation of granulocytes/neutrophils and inflammatory monocytes are elevated in the cornea of C3 KO mice at day 14 post infection. HSV-1 infected C3 KO and WT mice were euthanized by exsanguination at 14 days pi, and the corneas were removed and enzymatically processed to single cell suspensions. (**A**) Absolute number of CD45^+^CD11b^+^CD115^+^ Ly6G and Ly6C myeloid cell subpopulations that do or do not express CCR2. Panels below are representative flow plots of cell distribution in terms of high- and low-expressing Ly6G and Ly6C phenotypes. (**B**) Absolute number of CD45^+^CD11b^+^CD115^−^ Ly6G and Ly6C myeloid cell subpopulations that do or do not express CCR2. Panels below are representative flow plots of cell distribution in terms of high- and low-expressing Ly6G and Ly6C phenotypes. *p < 0.05 comparing the C3 KO to WT mouse CD45^+^CD11b^+^Ly6G^+^Ly6C^low^CCR2^+^CD115^+^ granulocyte/neutrophil phenotype, **p < 0.01 comparing the C3 KO to WT mouse CD45^+^CD11b^+^Ly6G^+^Ly6C^high^CCR2^+^CD115^+^ inflammatory monocyte, and *p < 0.05 comparing the C3 KO to WT mouse CD45^+^CD11b^+^Ly6G^−^Ly6C^low^CCR2^−^CD115^+^ immature granulocyte as determined by unpaired, two-tail *t*-test. The results displayed are from two independent experiments.

Components of the CS are potent chemoattractants for granulocytes and monocyte/macrophage populations^45^. As chemokines associated with recruitment of these cells including CXCL1 and CCL2 were also found to be increased in the C3 KO mouse cornea following HSV-1 infection, we next sought to determine whether changes in expression of chemoattractants altered the behavior of leukocyte recruitment. Corneas from WT and C3 KO mice were assessed for myeloid cell populations 3–14 days pi (Figs. 4, 5 and 6). As early as 3 days pi, there was an increase in CD11b^+^CD115^−^CCR2^−^Ly6G^+^Ly6C^low^ neutrophil/granulocyte population residing in the cornea of C3 KO mice (Fig. 4). No other myeloid cell population was found to be different. By day 7 pi, a number of different myeloid cell populations were elevated in the cornea of C3 KO mice including CD11b^+^CD115^+^CCR2^+^Ly6G^+^Ly6C^+^ inflammatory monocytes, CD11b^+^CD115^+^CCR2^+^Ly6G^−^Ly6C^low^ monocytes, and CD11b^+^CD115^+^CCR2^+^Ly6G^−^Ly6C^high^ macrophages as well as other myeloid cell populations ( CD11b^+^CD115^+^CCR2^−^Ly6G^+^Ly6C^low^, CD11b^+^CD115^−^CCR2^+^Ly6G^−^Ly6C^high^, CD11b^+^CD115^−^CCR2^−^Ly6G^−^Ly6C^low^ and CD11b^+^CD115^−^CCR2^−^Ly6G^−^Ly6C^high^ cells) (Fig. 5). By day 14 pi, there were still specific CD11b^+^CD115^+^CCR2^+/−^ myeloid populations elevated in the cornea of C3 KO mice (Fig. 6). However, the CD11b^+^CD115^−^CCR2^+/−^ myeloid populations had resolved in the corneas of WT and C3 KO mice with the exception of the CD11b^+^CD115^−^CCR2^+/−^Ly6G^+^Ly6C^low^ neutrophil population that were elevated in comparison to earlier time points (Fig. 6). Taken together, the overall myeloid-derived cell influx into the cornea of HSV-1-infected C3 KO mice is elevated and retained compared to the profile found in HSV-1 infected WT mice.

### Resolution of HSV-1 antigen is delayed in the cornea of C3 KO mice

**Figure 7:**
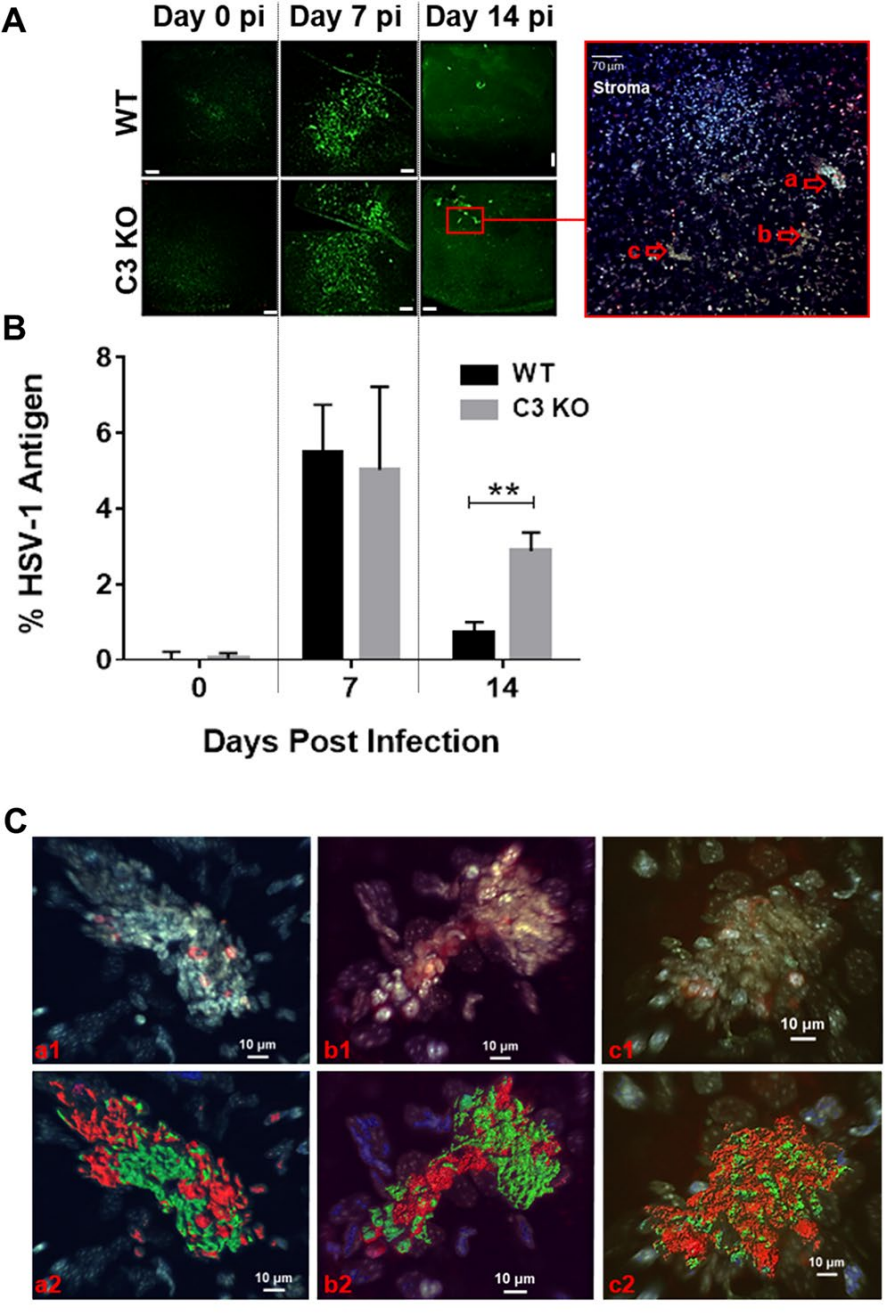
Clearance of HSV-1 antigen following acute ocular infection is hindered in C3 KO mouse corneas. HSV-1-infected WT and C3 KO mice were euthanized by exsanguination at day 7 or day 14 pi. Corneas were surgically removed and processed for immunohistochemistry using anti-HSV-1 and anti-CD45 antibodies as described in Materials and methods section. (**A**) Representative confocal microscopy images of flat mount cornea preparations from 0 (non-infected), 7 and 14 dpi of WT and C3 KO mouse corneas. HSV-1 antigen expression is displayed in the stroma in green. No antigen was detected in the epithelial layers. Magnification was 10× (*Z*-stack = 25 slices, 4.77 µm step size, Scale bar: 100 μm). Red square is an amplification of one particular region of C3 KO at 14 dpi, deeper focus was made to amplify the stroma excluding the corneal-epithelium (Z-stack = 40 slices, 1.77 µm step size, Scale bar: 70 μm). Right, three HSV-1antigen reactivity areas are highlighted (a, b and c). Cells in red are CD45^+^ and cells in green are HSV-1^+^ with DAPI staining in blue. (**B**) Metamorph quantification of the cornea area positive for HSV-1 antigen. Horizontal bars depict mean of percentage threshold area positive for anti-HSV-1 positive spots ± SEM (*p < 0.05 comparing the two groups as determined by unpaired, two-tailed t-test comparison, n = 3 per treatment, two repetitions). (**C**) Three dimensional reconstruction of stromal image in panel A highlighted by arrows a, b, and c are shown using IMARIS software depicted as a1, b1 and c1 and a2, b2 and c2 (Z-stack = 100 slices, 0.55 µm step size, Scale bar: 10 μm). Series 1 includes CD45, HSV-1 antigen, and DAPI stained profiles. Series labeled “2” includes CD45 and HSV-1 antigen staining. CD45^+^ cells are in green, HSV-1^+^ cells are in red, and blue is DAPI staining.

Following primary ocular infection, infectious virus is typically cleared within 10 days pi. However, as shown in this and previous studies^1,11,35^ an active immune response and progression of blood and lymphatic vessels occur in the cornea. Earlier work by several labs suggested HSV-1 DNA is retained in the cornea following lytic infection long into latency in experimental animal models and the human patient^46–49^. Therefore, we investigated HSV-1 DNA content in the cornea of infected WT and C3 KO mice over time. Viral DNA was readily detectable in the cornea of mice out to day 7 pi (data not shown). However, at later time points including days 14 and 21 pi, no HSV-1 DNA was detected in the cornea of either WT or C3 KO mice (n = 6/group/time point). As another means of explaining an ongoing immune response in the cornea following clearance of infectious virus, HSV-1 antigen expression was assessed at times post infection. Whereas no HSV-1 antigen was detected in uninfected WT or C3 KO mice, viral antigen was readily detected in the cornea of infected mice at day 7 pi (Fig. 7A). Similar to viral titers, there was no difference in expression of HSV-1 antigen at this time point comparing WT to C3 KO mice. By comparison, at day 14 pi HSV-1 antigen was still detected in the cornea stroma of WT and C3 KO mice with more antigen expressed in C3 KO mouse cornea (Fig. 7A,B). HSV-1 antigen was expressed in the stroma proximal to the epithelial layer of the cornea associated with and without CD45^+^ leukocytes (Fig. 7A,C). We interpret these results to suggest residual viral antigen may be responsible for the ongoing inflammatory response and soluble factor expression that contribute to the growth of blood and lymphatic vessels into the cornea following clearance of infectious virus under these experimental conditions.

## Discussion

Complement has been implicated in a number of ocular diseases including diabetic retinopathy, autoimmune uveitis, glaucoma, and age-related macular degeneration^50^. Viral pathogens including HSV-1 have evolved immune-evading mechanisms including those that target the CS^51^. Since the CS is composed of numerous proteins included in three different pathways that converge at the level of C3 in the activation process of downstream mediators^52^, the loss of C3 would address the role of the CS in HSV-1 ocular pathogenesis regardless of the pathway activated following infection. Previously, we found that the CS contributed to the efficacy of the HSV-1 0∆NLS vaccine against ocular infection in that HSV-1 0∆NLS vaccinated C3 KO mice possessed significantly more HSV-1 in the cornea and innervating trigeminal ganglia compared to vaccinated WT mice^4^. The loss of efficacy was also associated with an increase in the infiltration of select myeloid cells into the cornea during acute infection of vaccinated C3 KO mice^53^. In the present study, the hypothesis that the CS contributes to the inflammatory process associated with ocular HSV-1 infection was tested in mice. Mice deficient in the quintessential component of the CS, C3, were found to display an accelerated inflammatory response in the cornea as measured by neovascularization, opacity, inflammatory factor expression, and leukocyte infiltration. These results counter the anticipated outcome of the study. One of the products in the activation of C3 pathway is iC3b which has previously been shown to dampen the inflammatory response within ocular tissue through production of TGF-β2 and IL-10^54^. However, this mechanism of immunosuppression is linked to tolerance induction at the level of the antigen presenting cell rather than acute exposure to a viral pathogen. We earlier reported C3 KO mice maintain innervation of the cornea whereas WT animals undergo denervation during acute infection^29^. Corneal sensory fibers express substance P^6^, and substance P has been linked to corneal opacity and neovascularization^55,56^. Therefore, it is conceivable substance P released early in response to HSV-1 infection may contribute to the acceleration of inflammation observed in the cornea of the C3 KO mice.

In the present article, neovascularization was found to be accelerated in the cornea of C3 KO mice following HSV-1 challenge. This change correlated with the influx of inflammatory monocytes and macrophages that express CCR2 and CD115 antigens as well as CCR2^−^CD115^+^ granulocytes/neutrophils into the inflamed corneas. Previously, we reported depletion of CD115^+^ myeloid cells after the onset of infection and initiation in the progression of new vessel growth blocked corneal neovascularization consistent with the current results^31^. Pro-angiogenic factors associated with HSV-1-induced neovascularization and/or lymphangiogenesis include FGF-2, IL-1, IL-6, IL-17A, TNF-α, VEGF-A, and VEGF-C^2,11,57–60^. Cells thought to be a source of these factors include resident epithelial cells and fibroblasts, inflammatory monocytes/macrophages, neutrophils, and T cells^11,15,60–63^. These factors and cells likely act in concert with one another with VEGF-A critical in the early induction of neovascularization whereas other factors provide an additive or synergistic effect or replace the primary role of VEGF-A in the progression or maintenance phase of the angiogenesis process^11^. In the current study, we evaluated IL-1α, IL-6, FGF2, and VEGF-A and found VEGF-A levels correlated with the elevation in corneal vessel progression at day 14 pi while IL-1α levels were elevated at day 7 pi. VEGF-A and IL-1α are known to facilitate endothelial cell growth, migration, and generation of tube-like structures consistent with the findings reported herein^41,64,65^. Furthermore, VEGF-A has recently been shown to promote sympathetic nerve growth into the cornea during acute HSV-1 infection^62^. In this experimental design, IL-6 and FGF2 levels were not significantly elevated in the C3 KO mice compared to the WT animals, although there was a trend consistent with accelerated neovascularization and lymphangiogenesis. Similar to the change in pro-angiogenic factor expression, the expression of select chemokines including CCL2 and CXCL1 were elevated in the cornea of C3 KO mice corresponding to an increase in myeloid-derived cells.

In comparison to neovascularization, the pattern of corneal opacity was distinct. Specifically, opacity continued to climb in the cornea of C3 KO mice through day 21 pi whereas in WT animals, the peak of opacity occurred at 14 days pi and was noticeably lower at day 21 pi. As this presentation did not correlate with pathology that is associated with opacity including neovascularization or leukocyte infiltration throughout the time course of the study in either WT or C3 KO mice, it was next determined if collagen lamellae disruption was altered to a similar degree that would demonstrate the correlate to opacity. However, collagen denaturation peaked at day 7 pi in the C3 KO mice and resolved by day 21 pi whereas in WT corneas, collagen denaturation peaked at day 14 pi with resolution by day 21 pi similar to C3 KO animals. Consequently, corneal opacity cannot be attributed to any single event that transpired during ocular HSV-1 infection that were surveyed in the study. Additional factors that contribute to opacity that were not part of the analysis in the current study include edema, occurrence of anterior and posterior synechiae, surface epithelial dystrophy, the dynamics of the extracellular matrix and keratocyte crystallins^39,53,66,67^. We presume a combination of events over the course of infection is involved in the severity of HSV-1-induced opacity following cornea challenge.

In the human host, HSV-1 DNA has been found in the cornea of patients with herpes keratitis^68^. The severity of the disease as well as the age of the patient was directly correlated with the prevalence HSV-1 DNA which has also been previously noted^69^. In the current study, we were unable to detect HSV-1 DNA following clearance of the virus but did observe foci of HSV-1 protein in the stromal layer that was more commonly found in C3 KO mouse corneas. Such results are consistent with the role of C3 and downstream fragments that assist in the phagocytosis of complement-bound viral antigens^70^. The existence of antigen in the tissue would maintain a local immune response which is exactly what was observed in the present study with leukocyte infiltration and cytokine/chemokine production. The observation that more antigen exists in the cornea of HSV-1-infected C3 KO mice compared with WT animals is consistent with the heightened immune profile and some aspects of pathology of the corneas at the day 14 pi time point. Differences observed at earlier time points would not be related to viral antigen per se but rather reflects the impact of the CS on inflammation in response to local virus infection and the complexity in the CS components in early regulation of cornea inflammation and neovascularization^71^.

In summary, the results showing the CS is a necessary component involved in reducing inflammation and some aspects of corneal pathology following HSV-1 infection was unexpected given the highly inflammatory properties of components of the CS including C3a/b and C5a^50,70^. It would seem that CS participation in viral antigen clearance trumps some aspects of inflammation in this unique and normally immunologically-privileged tissue^72^. Since HSV-1-induced corneal pathology is layered within components of the innate and adaptive immune response, it will be challenging but necessary to further identify those pathways including cells and soluble factors that contribute to disease and thus, may serve as candidates to target for therapeutic intervention.

## Materials and methods

### Mice

C3 KO mice (stock number 003641 on a C57BL/6 background expanded as a colony at the Dean McGee Eye Institute vivarium) and C57BL/6 mice (stock number 000664) were obtained from The Jackson Laboratory (Bar Harbor, ME). Male and female mice were between 7 and 10 weeks of age at the time of performing experiments. Prior to scarification, harvesting tissue, or euthanizing the animals, mice were anesthetized with ketamine (100 mg/kg) and xylazine (6.6 mg/kg) intraperitoneally and exsanguinated by cardiac perfusion with 10 mL of PBS. All animal procedures as described in the Methods section were approved by the University of Oklahoma Health Sciences Center Institutional Animal Care and Use Committee (protocol #19-008-AI) and were performed in adherence to the Association for Research in Vision and Ophthalmology Statement for the Use of Animals in Ophthalmic and Vision Research. This study was carried out in compliance with the ARRIVE Essential 10 Guidelines.

### Virus and infection

HSV-1 strain McKrae was propagated in green monkey kidney (Vero) cells and maintained at a stock concentration of 1.7 × 10^8^ to 1.7 × 10^9^ plaque-forming units (PFU)/mL. Anesthetized mice were infected by scarification of the corneal surface with a 25-G 1½” needle and tear film blotted, followed by application of 2.0 µL of RPMI medium containing virus (500 PFU/eye) as previously described^17^. Non-infected control animals included scarification of corneas only.

### Tissue removal, dissection, and viral plaque assay

Using aseptic technique, the eyes, TG, and brains were removed from exsanguinated mice. The corneas were removed under a Zeiss Stemi DV4 dissecting microscope (Carl Zeiss AG, Oberkochen, Germany) and placed in 0.5 mL RPMI-1640, 10% FBS, and antibiotic/antimycotic solution (all from Invitrogen, Grand Island, NY) (complete media) per cornea pair. TG were also added to 0.5 mL complete media per TG mouse. Brain regions including the brain stem, hippocampus, midbrain, and subventricular zone were dissected on an iced surgical block. Isolated regions were added to 0.5 mL complete media. The tissue was homogenized using a tissue-tearor (Dremel, Racine, WI) on ice, then centrifuged at 10,000×*g* for 1 min at 4 °C. The clarified supernatant was serially diluted in complete media and added to Vero cell monolayers for 1 h in 96-well plates and then discarded and replaced with 100 µL complete media containing 0.5% methyl cellulose. Plates were read for plaque formation 30–42 h later.

### Immunohistochemistry and confocal microscopy

Corneas were removed after exsanguination of uninfected mice or mice at various time points pi. The corneas were fixed in 4% paraformaldehyde (w/v in PBS) for 30 min at room temperature (RT). Tissue was then incubated overnight at 4 °C with gentle agitation in 1 mL 1% Triton X-100 in PBS per cornea pair. Non-specific sites were blocked overnight at 4 °C with gentle agitation in 10% normal donkey serum in 0.1% Triton/PBS. The corneas were then washed at RT 3× (20 min each) with gentle agitation in 0.1% Triton/PBS. Primary antibody including Armenian hamster anti-mouse CD31 (Millipore, Billerica, MA), rabbit anti-mouse LYVE-1 (Abcam, Cambridge, MA), rabbit anti-HSV1-FITC conjugated antibody (Dako Agilent, Santa Clara, CA), rat anti-mouse CD45 (BD Pharmigen, San Diego, CA) in 1:200 dilution in 0.1% Triton/PBS was added followed by overnight incubation at 4 °C with gentle agitation. The corneas were then washed at RT 3× (20 min each) with gentle agitation in 0.1% Triton/PBS. Secondary antibody was then added and included goat anti-rat IgG conjugated with Alexa Fluor 546 (Invitrogen, Eugene, OR), donkey anti-rabbit Alexa Fluor 488 (Jackson ImmunoResearch, West Grove, PA), goat anti-hamster TRITC (Jackson ImmunoResearch) all diluted 1:200 in 0.1% Triton/PBS and incubated overnight at 4 °C with gentle agitation. Following the washing steps, 40, 6-diamidino-2-phenylindole (DAPI diluted 1:10,000 in PBS) was added for one hour at RT. The corneas were then mounted in 50% glycerol in PBS and imaged with an Olympus laser scanning confocal microscope, FV1200 (Olympus Corp., Center Valley, PA). The total area positive for vasculature or HSV-1 antigen per field of view (4 quadrants/cornea) was quantified using Metamorph software (version 7.7.0.0; Molecular Devices LLC, Sunnyvale, CA). Three dimensional rendering of cornea images was accomplished by collecting *z*-stack images of the stained areas using the Olympus FV1200 confocal microscope with a step size of 0.5 µm and a numerical aperture of 1.25 at a magnification of 400×. The images were processed to create 3D deconvolution using IMARIS software (Bitplane Inc., Concord, MA).

### Corneal opacity measurement and collagen stain

This process has previously been described with modifications in the present study^31^. Specifically, corneas were harvested from infected or uninfected C3 KO and WT mice and fixed for 30 min at RT in 4% paraformaldehyde (^w^/_v_ in PBS), followed by washing 3×, 20 min per wash in 1 mL PBS. Corneas were cut into four quadrants and placed in the flat-bottom of a 96-multiwell (one cornea per well). To flatten the corneas, one glass circle of 5 mm diameter was placed on the top of each cornea. PBS was gently added (5 μL). The tissue was assayed for absorbance at 500 nm in the FLUOstar Omega (BMG LABTECH, Offenburg, Germany). To measure denatured collagen strands, corneas were permeabilized, washed, stained and measured as previously described^31^. Briefly, corneas were permeabilized as described above and placed in the bottom of the well of a 150-well Microtest Plate (Greiner BIO-ONE, Monroe, North Carolina). The wells were then placed on ice, and 20 μL of 20-μM biotinylated collagen hybridizing peptide (B-CHP; 3Helix, Salt Lake City, UT, USA) was added to each cornea followed by overnight incubation at 4 °C. Corneas were then washed three times in 1 m PBS at RT (10 min per wash) and incubated overnight at 4 °C with Alexa Fluor 647 conjugated with streptavidin (3.85 μg/mL; Jackson ImmunoResearch, West Grove, PA). Prior to mounting, the corneas were washed as before. The corneas were then mounted in 50% glycerol and imaged using an Olympus FluoView confocal laser scanning microscope (Olympus Corporation).

### Spectral domain-optical coherence tomography and esthesiometry

Prior to (uninfected) and following HSV-1 infection, the corneas were imaged using a Bioptigen spectral domain-optical coherence tomography (SD-OCT) system (Leica Microsystems, Buffalo Grove, IL) to measure cornea edema and inflammation. The blink reflex (cornea mechano-sensory function) was assessed on non-anesthetized uninfected and infected WT and C3 KO mice using a Luneau Cochet-Bonnet esthesiometer (Western Ophthalmics, Lynwood, WA) as previously described^6^.

### Soluble protein extraction and multiplex suspension array

Corneas and TG were harvested and placed in sterile PBS containing protease inhibitors cocktail set 1 (EMD Millipore, Bellerica, MA) in 250 μL/cornea pair. Tissue was homogenized using 1.5 mL snap-capped Eppendorf tubes (Advanced Bullet Blender, Troy, NY) and Bullet Blender Storm 24 (Advanced Bullet Blender) three times for 5 min each at the maximum setting, with centrifugation at 10,000×*g* between each round. Finally, samples were centrifuged (1 min, 10,000×*g*), and the protein content in the clarified supernatant was determined using a Pierce BCA Protein Assay Kit (Thermo Fisher Scientific, Waltham, MA). The protein concentration of IL-1α, CXCL-1, CCL2 and VEGF-A were determined using Mouse Magnetic Luminex Assays (BioRad Laboratories, Hercules, CA, USA). Metalloproteinase (MMP)-2, MMP-3, and MMP-8 levels were determined using Mouse Magnetic Luminex Assays (EMD Millipore). Samples were assessed using a BioPlex 200 System (Bio-Rad). The concentration of each candidate protein is expressed as pg analyte/mg soluble protein.

### Flow cytometry

To obtain single cell suspensions, the extracted corneas harvested from C3 KO and WT mice at times pi were incubated with Liberase TL (Roche, Mannheim, Germany) for 30 min at 37 °C. Digested tissue was filtered through a 40-μm cell strainer and washed with staining buffer (PBS supplemented with 2% FBS). Single cell suspensions were treated with CD16/CD32 Ab (clone 93) for 10 min at 4 °C to block nonspecific binding and next stained for 20 min on ice with the combination of fluorochrome-conjugated anti-mouse antibodies (all from Biolegend, San Diego, USA): anti-CD45 pacific blue conjugated, anti-CCR2/CD192 FITC or BV650 conjugated, anti-MHCII (I-A/I-E) APC-Cy7 conjugated, anti-Ly6C APC conjugated, Ly6G PerCP-Cy5.5 conjugated, anti-CD115 PE-Cy7 conjugated, and anti-CD11b PE conjugated. Data were acquired on either a MacsQuant flow cytometer (Miltenyi Biotec, Auburn CA, USA) or spectra flow cytometer Aurora (Cytek Biosciences, Fremont, CA USA) and analyzed with FlowJo software (FlowJo LLC, Medford, OR). Representative flow charts are presented along with the summary for each antibody combination that has identified cell populations.

### Statistical analysis

Data are presented as mean ± SEM. Prism 5 software (GraphPad, San Diego, CA, USA) was used for statistical analysis, and tests utilized are described in each figure legend. Data were considered to be significant at *p* < 0.05.

## Supplementary Information

Supplementary Information.

## Data Availability

The datasets generated during and/or analyzed during the current study are available from the corresponding author on reasonable request.
