# Supplementary material for: An intact complement system dampens cornea inflammation during acute primary HSV-1 infection
Source: Sci Rep. 2021 May 13;11:10247. doi: 10.1038/s41598-021-89818-9 (PMC8119410; doi:10.1038/s41598-021-89818-9)
Supplement: Supplementary file 1 — Supplementary Information. [file 41598_2021_89818_MOESM1_ESM.pdf]

Supplementary Figure 1

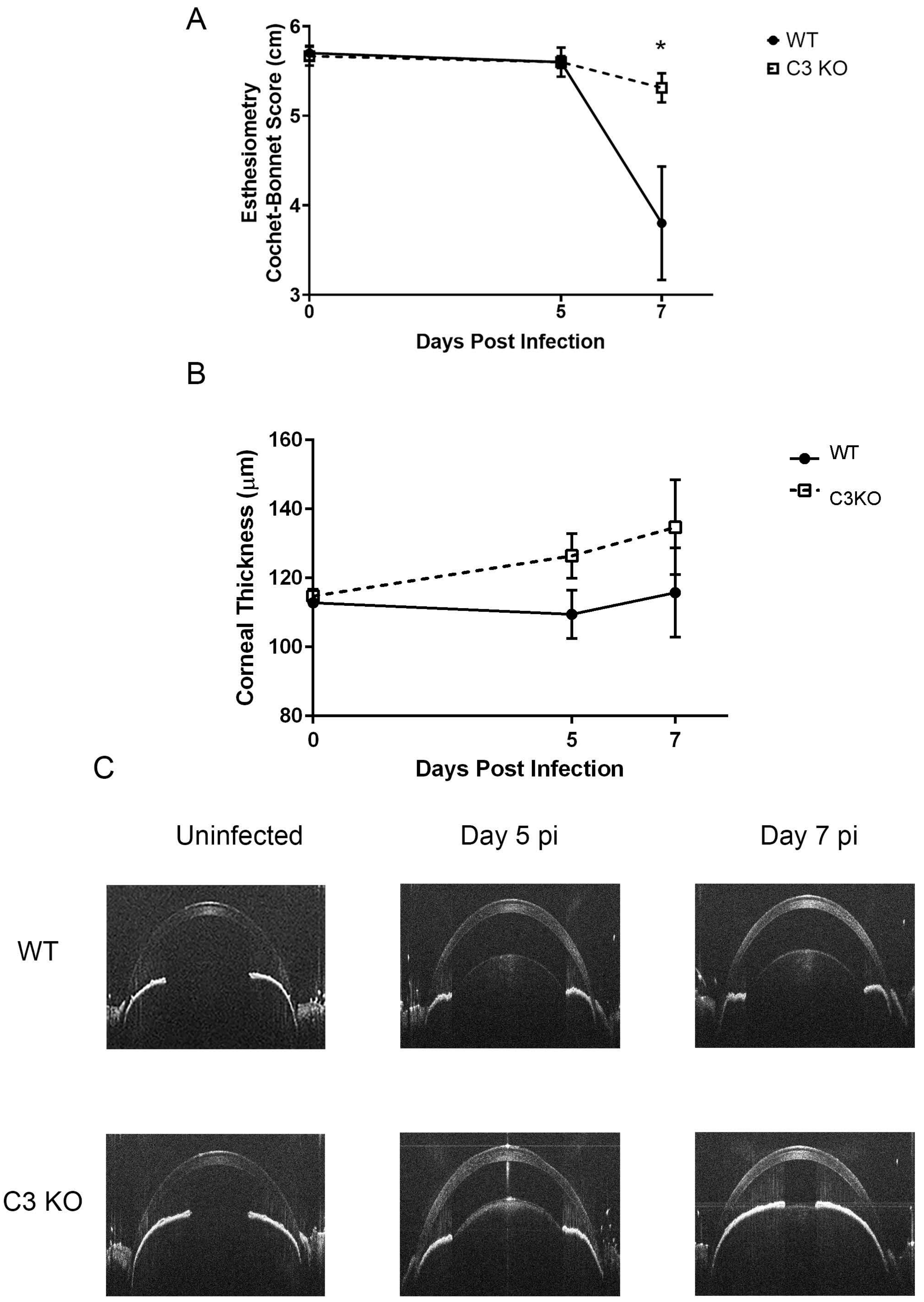

Supplementary Figure 1. C3 KO mice display no loss in mechanosensory function but possess an increase in cornea edema and inflammation compared to WT mice following ocular HSV-1 infection. Wild type (WT) and complement component 3 deficient (C3 KO) mice (n=5/group) were infected with HSV-1 (500 pfu/eye). (A) Prior to (0) and at day 5 and day 7 post infection (pi) mouse corneas were assessed for mechanosensory function using a Cochet-Bonnet esthesiometer. Uninfected mice served as the control for baseline measurement. The results are expressed as the mean ± SD. *p<.05 as determined by two-way ANOVA and Sidak's multiple comparison test. (B) Immediately after the esthesiometry measurements were conducted, the mice were anesthetized and spectral domain-optical coherence tomography (SD-OCT) analysis was conducted on the corneas of the sedated animals at times prior to or post infection. The results are expressed as the mean ± SD. (C) Representative images obtained from SD-OCT are shown for uninfected as well as day 5 and day 7 pi corneas of WT and C3 KO mice.
